# Tools to overcome potential barriers to chlamydia screening in general practice: Qualitative evaluation of the implementation of a complex intervention

**DOI:** 10.1186/s12875-016-0430-2

**Published:** 2016-03-22

**Authors:** Ellie J. Ricketts, Elaine O’Connell Francischetto, Louise M. Wallace, Angela Hogan, Cliodna A. M. McNulty

**Affiliations:** Public Health England Primary Care Unit, Microbiology Department, Gloucestershire Royal Hospital, Great Western Road, Gloucester, GL1 3NN UK; NIHR CLAHRC West Midlands Chronic Disease Theme, Institute of Applied Health Research, University of Birmingham, Edgbaston B15 2TT, Birmingham, UK; Health Protection Agency Primary Care Unit, Personalised Medicine Consortium Integrated Biobank of Luxembourg 6, Rue Nicolas Ernest Barblé, L-1210 Luxembourg; Faculty of Health and Social Care, National Institutes of Health Research Health Services and Delivery Research Programme, The Open University, Walton Hall, Milton Keynes, MK7 6AA UK

**Keywords:** Chlamydia screening, General practice, Primary care, Normalisation process theory, Education, Sexual health

## Abstract

**Background:**

*Chlamydia trachomatis* remains a significant public health problem. We used a complex intervention, with general practice staff, consisting of practice based workshops, posters, computer prompts and testing feedback and feedback to increase routine chlamydia screening tests in under 25 year olds in South West England. We aimed to evaluate how intervention components were received by staff and to understand what determined their implementation into ongoing practice.

**Methods:**

We used face-to-face and telephone individual interviews with 29 general practice staff analysed thematically within a Normalisation Process Theory Framework which explores: 1. *Coherence* (if participants understand the purpose of the intervention); 2. *Cognitive participation* (engagement with and implementation of the intervention); 3. *Collective action* (work actually undertaken that drives the intervention forwards); 4. *Reflexive monitoring* (assessment of the impact of the intervention).

**Results:**

Our results showed *coherence* as all staff including receptionists understood the purpose of the training was to make them aware of the value of chlamydia screening tests and how to increase this in their general practice. The training was described by nearly all staff as being of high quality and responsible for creating a shared understanding between staff of how to undertake routine chlamydia screening.

*Cognitive participation *in many general practice staff teams was demonstrated through their engagement by meeting after the training to discuss implementation, which confirmed the role of each staff member and the use of materials. However several participants still felt unable to discuss chlamydia in many consultations or described sexual health as low priority among colleagues. National targets were considered so high for some general practice staff that they didn’t engage with the screening intervention.

*Collective action *work undertaken to drive the intervention included use of computer prompts which helped staff remember to make the offer, testing rate feedback and having a designated lead. Ensuring patients collected samples when still in the general practice was not attained in most general practices.

*Reflexive monitoring *showed positive feedback from patients and other staff about the value of screening, and feedback about the general practices testing rates helped sustain activity.

**Conclusions:**

A complex intervention including interactive workshops, materials to help implementation and feedback can help chlamydia screening testing increase in general practices.

## Background

Genital *Chlamydia trachomatis* is the most common sexually transmitted infection reported in Europe and rates continue to rise [1]. The European Centre for Disease Prevention and Control [2] stressed the need for effective diagnosis and treatment of symptomatic chlamydia and active case-finding to detect and treat asymptomatic cases. Rates are high within England [3] and remain a key public health challenge [4] in order to reduce transmission and the public health impact of complications arising from untreated disease on adult reproductive health and neonates.

The delivery of testing within primary care is a key aim of the National Chlamydia Screening Programme [5] as the majority of young adults visit their general practice at least annually [6]. Young people have expressed preferences to be offered a chlamydia screen at their general practice rather than in other venues [7]. Once primary care patients have had a chlamydia test they are more likely to test again, are aware of how to avoid infection and have fewer sexual partners [8].

Detailed guidance is available to encourage in general practice testing [5, 9] of patients under 25 years, as this target group bears the largest burden of disease. General practice staff report barriers to offering chlamydia screening, including competing priorities, not receiving test results or feedback on screening rates and a lack of normalcy around chlamydia screening [10–13]. Awareness of barriers and overcoming them is viewed as a key strategy in the successful implementation of evidence based guidance in healthcare setting [14–16] but facilitators are less frequently identified [17].

Normalisation Process Theory (NPT) [18] provides a framework for understanding the collective processes by which complex interventions are implemented, embedded and integrated into everyday work, and sustained in practice. This is appropriate since the intervention is focussed at general practice level, as opposed to individual practitioner behaviour. NPT uses four elements which interact to allow a deeper understanding of how a new intervention (in this case chlamydia screening of under 25 s) can be adopted into routine general practice. 1. *Coherence* concerns individual and group clarity regarding the detail and purpose of the intervention and their role within it. 2. *Cognitive participation* describes mental engagement with the work and undertaking action on an ongoing basis to maintain it. 3. *Collective action* incorporates all the work actually undertaken that drives the intervention forwards and reinforces belief in its effectiveness. 4. *Reflexive monitoring* is the process whereby the individual and group assess the impact of their work and make adjustments based on this. Understanding what was important for staff in embedding a new intervention in general practice will assist people interested in utilising an intervention model and implementing it into new settings, by illuminating the cognitive and practical processes staff undergo. How barriers are addressed provides important insight into translational process.

### The Chlamydia Intervention Randomised Controlled Trial (CIRT)

We have previously reported the effect of a complex intervention that aimed to address reported barriers to chlamydia screening within general practices [19]. The complex intervention was based on the Theory of Planned Behaviour and aimed to address personal attitudes, subjective norms and behavioural controls that were influences on intention of general practice staff opportunistically offering chlamydia screening to all 15–24 year olds visiting their general practice. Between April and September 2010, all intervention surgeries were offered a general practice visit from a Chlamydia Support Worker (CSW) employed by the Health Protection Agency. At this visit the CSW gave a presentation to as many staff as possible (including general practitioners, nurses, managers and receptionists) that aimed to change intention to offer chlamydia screening. The workshop covered the epidemiology of chlamydia infection and the public health benefits of diagnosing asymptomatic and symptomatic infections. The general practice’s population and consultation figures were used to demonstrate how many patients aged 15–24 years were registered and attended the general practice, and so developing realistic targets for screening and how many offers each member of staff should aim to make per week or month. Perceived behavioural control in offering chlamydia screening was addressed through videos modelling how an offer could be made in different patient consultations, and provided staff with a script to use. The CSW also offered materials and interventions addressing subjective norms including posters and patient invitation cards for receptionists and others to give out to patients in the at risk age group to raise awareness of screening to staff and patients. Participating general practices were sent bimonthly newsletters feeding back their practice results and updates about chlamydia. The CSW identified a “chlamydia champion” in each general practice who maintained the profile of screening within that general practice. Forgetting to offer screening was addressed through the CSW asking staff to add chlamydia to appropriate computer templates (such as travel or contraception) and/or add a pop up prompt for all 15–24 year old patients. The general practice staff could accept or refuse any part of the intervention at any time during the study period and the CSW worked with general practice staff to adapt the intervention to fit in with the general practice premises, computer system and culture. During the 9 months after the first visit, the general practice was offered two further visits or telephone calls, to discuss how the general practice was implementing the materials, offered further support, and training. The intervention was found to be effective in increasing the screening rate of general practices in the study [19]. A further newsletter was sent to participating general practices in August 2011.

We report a qualitative process evaluation of the intervention. We aimed to determine how the intervention was experienced by general practice staff and understand why general practices did or did not adopt them. We sought to explore individual and general practice level behaviour change, and determine whether their intentions to offer chlamydia screening had increased and if there were any remaining barriers to chlamydia screening.

## Methods

The 78 CIRT intervention general practices were stratified by their chlamydia screening rates over the six months after they attended workshops. Screening rates were determined using routinely generated National Chlamydia Screening Programme data. They were divided into centiles by their increase in screening rates compared to the year before the intervention period, and the third of practices with the highest and lowest centile change in testing were randomised. All staff who had participated in the workshop were approached to take part; this included general practitioners, nurses and receptionists. Each stratified group was approached in random order by invitation letter and telephone call. Participation involved an interview to discuss their opinions on the support they had received from a CSW. They were unaware their practice was part of a randomised controlled trial; a modified McNulty-Zelen design [19–21] was used with consent given by appropriate Primary Care Trust leads for general practices in South West England to be randomised to the trial without their knowledge. Interview participants gave informed consent to be interviewed and for interviews to be recorded, transcribed and for anonymised data to be used in publications. Interviews were conducted by researchers not involved with the delivery of the intervention and continued until there were no additional themes emerging from the interviews and data saturation was reached. Interviews took place 6–8 months after the intervention ended.

The interview schedule covered the intervention’s impact on both the interviewee’s and whole general practice’s approach to chlamydia testing and the feasibility of the intervention being used by other general practices (Appendix). Interviews were conducted in person or by telephone. Staff could choose to not answer any of the questions; indeed several of the receptionists felt unable to answer most of the questions as they had no involvement in chlamydia screening. Interviews ranged in length from 7 to 43 min. Interviews were digitally recorded, transcribed and checked for accuracy.

### Analysis

Transcripts were read and reread to develop a deep familiarity with them. An initial inductive thematic analysis was undertaken on the first 10 transcripts by two researchers (ER/EOF) independently reviewing the data for emerging themes; the two researchers reported similar themes. They then discussed these codes and agreed a draft framework [22]. After this initial inductive analysis all further interviews were undertaken, and interviewers probed to ensure that these initial themes were explored. The second stage of the analysis involved a theoretical thematic analysis approach. The framework developed in the first stage was refined and the four constructs of NPT were used to further investigate emerging themes across the entire set of data [18, 22]. In this second stage ER analysed all of the transcripts and EOF analysed 40 % to ensure the codes and themes developed from this second stage were fully representative of the data.

## Results

Participants included 9 general practitioners, 13 nurses and 7 receptionists from high (8) and low (7) screening intervention surgeries Table 1.

### COHERENCE (was the purpose of the intervention to increase chlamydia screening and thus reduce chlamydia infections, onward transmission and sequelae clear?) Table 2

The training was described by nearly all staff as being of high quality and responsible for creating a shared understanding between staff of how to undertake routine chlamydia screening and why it was important. Only one participant, a receptionist working in a small general practice premises where screening was perceived as very difficult due to lack of confidentiality in the reception area, said she would not recommend it to other general practices or commissioners of sexual health services.

All staff remembered that the training purpose was to make them aware of the prevalence of chlamydia, the target group for screening and that screening is required to identify infection, prevent transmission and potential sequelae.

Most participants said it was important that all general practice staff were involved, so they understood each other’s work and agreed an approach that worked for their general practice. Most of the receptionists thought that screening was a good idea and the training was useful for them so that they were aware of what was involved, even if they themselves were not inviting patients to participate because of confidentiality issues within the reception area.

Barriers were mentioned less frequently than positive feedback about the training and positive experiences of implementing the intervention. There were trivial differences between the 3 staff groups. Barriers were mentioned more frequently by staff at low screening general practices but did not differ in content. Staff in the high screening general practices overcame the barriers as they were more committed to screening, this was partly due to the training but also that some of the practitioners and general practices had a greater interest and belief in sexual health. Only low screening general practices reported, despite the training, believing that it was the patient’s responsibility to ask for a screen.

### COGNITIVE PARTICIPATION (Staff engaged in the training and their role in implementation in practice) Table 3

When asked about the general practice approach to screening, participants reported that most staff teams met to discuss implementation, which demonstrates engagement in the training, and confirmed the contribution of each staff member in attaining the purpose of the intervention. As a result most participants reported that general practice staff undertook a variety of recommended actions that supported embedding routine screening. This included establishing computer prompts for a variety of consultations e.g. travel vaccinations, contraception and the screening age group of 15–24 years. It included displaying posters and half placed screening kits in consultation rooms, reception desks and sometimes toilets. In low screening practices, the prompts and posters to optimise screening were less likely to be remembered by staff and enacted.

Since the training most staff reported that they had changed how they made an offer to patients, and most narrated the phrases they now used which were based on the phrases used by the trainers. When participants were asked about how confident they felt to offer chlamydia screening and what made it easier, about half of participants reported that the training had made them more aware that patients wanted general practice staff to offer them chlamydia screening and nearly all reported the training had increased their confidence to offer it. Ensuring the test was done by the patient before leaving the general practice was said to be important by some.

Staff in high screening general practices (in contrast to low) tended to describe adopting and adapting the intervention, even where they did not like particular elements, or found them difficult to adopt, because they believed the work important. A few high screening general practices used extra interventions not recommended within CIRT in an attempt to further increase screening; this included writing to all patients in the target age group to invite them for screening, or placing screening kits in the toilets. These actions were reported as unsuccessful.

### Barriers to cognitive participation

A few of the participants’ own views were a barrier to increasing chlamydia screening in their practice. They saw it as not always appropriate to raise chlamydia screening; if a parent was present, if the patient was not consulting about sexual health, or if it didn’t ‘feel’ right. Despite the training stressing that young people welcomed being offered screening, two participants were concerned that patients would be irritated by the offer. One practitioner thought that the national target of screening 35 % of 15–24 year olds set by the National Chlamydia Screening Programme was too high for their rural area, which he thought would have fewer chlamydia infections. This led to him to not making any effort to reach the target, and this general practice tested less than 1 % of 15–24 year olds. A few participants described sexual health as low priority among general practice colleagues.

### COLLECTIVE ACTION (work undertaken to drive the intervention forward and sustain it) Table 4

Intervention components designed to focus the whole general practice’s attention on chlamydia screening were frequently reported as successful. Computer prompts were reported by many as important in helping staff remember to make the offer. In a university practice the receptionist reported that they gave out information about screening availability in the practice, as all new students registered at the practice, and also gave out chlamydia kits (usually at a side window “*where you can be a little bit more discreet about things*.”)

The value of having a “chlamydia champion”, most often the practice nurse, for tasks such as organising general practice’s processes to support screening was acknowledged. Further contact with the CSW also supported screening practice.

### BARRIERS: (to collective action)

A few nurses and one receptionist reported that they were unable to influence implementation as the general practice partners determined which areas of activity were prioritised; this was a significant barrier to either putting the intervention components in place, or the intervention being adopted by the staff team as a whole.

Elements of CIRT were sometimes not adopted because they conflicted with the usual general practice systems. Most frequently because of time issues staff did not ensure patients completed the screening tests before they left the practice. Despite indicating intention and technical competence at the initial training to develop computer prompts to remind staff to undertake screening in all consultations with 16–24 year olds, some general practices did not follow through with setting up the prompts. One practice did not use the posters as they were worried that patients may complain, and a few had not used the invitation cards as they thought the reception area lacked confidentiality. Participants reported that these reasons for non-adoption discouraged them from continuing to offer tests.

Receptionists were mentioned by some participants as not forming part of the general practice’s efforts. The reasons included belief that their involvement was inappropriate due to workload, would compromise patient confidentiality, their lack of confidence or lack of further support within the general practice to embed their screening role. Receptionists themselves were divided in their views. One receptionist actively gave out leaflets and kits to new students registering, and another gave out kits that were placed at reception, a third had been trained but was not happy about confidentiality and therefore was not actively promoting screening in the reception area. Only one receptionist was against being involved in the training. Two were supportive of screening, but thought that due to perceived confidentiality issues in the reception area and constraints on their time it was better managed by nurses or doctors. Two nurses using telephone triage found it more difficult to broach screening without prompts that normalise it, e.g. posters in the room they could refer to. The practicalities of maintaining a supply of in-date screening packs was a barrier for some participants.

### REFLEXIVE MONITORING (appraisal of the new procedures and practices in use and its impact, and making refinements) Table 5

A variety of positive responses from patients encouraged participants to continue their changes in practice. Participants cited patient familiarity with the testing process and acceptance of the offer improved their confidence that it was welcome. Participants felt it improved their patient relationships, and facilitated sexual health discussion in general.

Commitment to sexual health work and chlamydia screening in particular was expressed and a positive approach to innovation or overcoming problems was described as important in maintaining screening efforts. Participants felt offering chlamydia screening to patients within routine consultations could be done very briefly, and efficient practitioners could absorb it into their practice without undue effect.

Receiving feedback on a monthly basis about numbers of tests performed was reported by many as important in motivating them personally to offer screening.

### BARRIERS to reflexive monitoring

Participants decided it was too time consuming to routinely offer chlamydia screening and some reported attitudes within the general practice that were unchanged.

## Discussion

This study indicates that the CIRT intervention can increase staff intentions to offer chlamydia screening and can lead to changes in general practice routines that facilitate it.

The training session was well received by the participants, and their views categorised under all four NPT elements reflect their use of the intervention in their practice, and, importantly, that of the general practice as a whole. The intervention influenced coherence of knowledge about the public health rationale for screening within the target group. They found that in the training session the discussion of the range of patient views and being offered a standard opening script was important in gaining their engagement, and raising practitioner confidence to make a screening offer. Receiving training as a general practice team fostered group understanding of the screening pathway and facilitated whole team agreement on adaptations to suit the organisation of screening in their general practice. Most receptionists were generally supportive of the training and screening, but although a few were actively involved in giving out kits, the majority were constrained by concern about confidentiality issues in the reception area.

The use of physical resources (posters, computer prompts and feedback of screening rates) helped create an expectation by the general practice staff of readiness for the entire target group to be approached. Conversely, where attitudes of other general practice staff to screening were perceived to be unaltered, or influential members of the general practice did not attend training, it was more difficult for staff to make changes.

Positive reflections by patients, other general practice staff and feedback on their screening practice were reported to be instrumental in embedding chlamydia screening tests into general practice. Participants reported that when general practice staff found screening offers did not create additional time burdens or adverse reactions by patients, screening gradually became routine practice.

### Intervention components facilitating normalisation of chlamydia screening into general practice

To promote whole general practice team engagement, at least one influential member of the general practice was needed to attend the intervention training to ensure that colleagues present also had further opportunities to maintain team engagement. It was important that other staff members understood their role in implementing screening tests (e.g. ensuring patient undertakes the test in the premises).

### Strengths and limitations

The modified McNulty-Zelen design of the original CIRT ensured that staff were unaware they were in a trial. Staff implemented the intervention as they would in normal practice. Interviews were conducted by staff that had no involvement with the delivery of the intervention, reducing the risk of social desirability bias. While participants may have forgotten some details of the intervention by the time interviews took place 6 months after the workshops, we considered that the most memorable components of the workshops would be recalled and reported. Participants who reported that they tended to incorporate changes in practice soon after the workshop, were more likely to remember components they were currently using in their daily practice and therefore the results are more relevant to the normalisation of the intervention. Furthermore, they should be able at 6 months after the workshop to give a clear indication of what had been implemented, as it is unlikely that components would be implemented after this time period. A limitation is that we only interviewed staff who were involved in the training; the work may have been strengthened by undertaking focus groups which included other staff so that we could explore with the whole general practice team if and how the training was disseminated. Focus groups would also allow us to explore further any remaining barriers and how successes were, or could be, exploited. We excluded medium screening rate general practices as we wished to explore the differences between successful and unsuccessful general practices. This may have led to us missing some themes arising from medium general practices. However our previous work suggests that barriers in medium and low general practices are similar [11, 12]. We encouraged face to face interviews in the work place, which allowed interviewers to understand the general practice environment. Offering telephone interviews as an alternative allowed us to interview more busy general practice staff (who reported they had insufficient time for a face to face interview) as it is less of an intrusion into their daily workload and easier as an interview room was not needed. This allowed researchers to meet participants’ preferences, and some participants may feel that they can be franker in a telephone interview [23]. Like others, we found that the themes arising from telephone and face to face interviews were very similar [23]. Some interviews were very short, this was because some of the receptionists, even though they attended the training, were not involved in chlamydia screening and therefore felt unable to comment further. Further work is needed to explore how receptionists with training could be involved in public health initiatives in the general practice setting, as the involvement of receptionists was very varied in this study with some fully involved in new patient checks and handing out cards, and others thinking their involvement was inappropriate.

As other researchers have found, there are challenges in differentiating the four elements of the NPT framework [24]. The use of NPT in implementation research is rapidly expanding. In future studies we would recommend having an independent researcher to verify the coding and analysis.

### Comparison with existing literature

A review of studies aiming to increase screening in primary care found those that promoted an offer by primary care staff through the routine provision of an offer of a test were most effective in raising screening rates [25]. Our study concurs with these findings as staff reported that receiving training in how to make a routine offer increased their confidence to do so, and the visual posters and computer prompts they received further supported this. An ethnographic study of knowledge management in primary care [26] found adoption of guidance was not achieved through reading it, rather it became embedded over time through interactions with clinical colleagues and adding it to existing knowledge. This gradual immersion into practice was a feature of our findings. Staff reported reflective discussion of their practice with colleagues, and being prompted to make offers by feedback on their performance and by computer and email reminders. The quantitative data from this study (reported elsewhere) [19] confirms the importance of computer prompts in embedding screening behaviour into practice, as those general practices (54 % of those who had a workshop) who used computer prompts, had a greater increase in screening rates (2.8 times) compared to controls, and these rates remained twice as high in the post-intervention period. Previous research has found that reminders may prompt doctors to remember information and subsequently have the potential to improve care [27]. Carlsen et al. [28] conclude flexibility in implementing guidance is important in recognition of the range of how patients present. We found where staff discussed how to fit the intervention with their particular workplace it strengthened their willingness to gradually adopt screening into daily work. Chenot et al. [29] reported in their study of adoption of new guidance into general practices, that simply giving the guidelines is not sufficient in affecting routine behaviour change by clinicians, even where they agree with the guidance. Existing beliefs, attitudes and physical barriers effectively stifle the adoption of the necessary thinking and small actions that in combination enact a physical manifestation of the guidance. Our study showed that the intervention implementation faltered where staff struggled to overcome existing barriers in the form of perceived negative staff attitudes of influential colleagues, or difficulties in the operation of screening pathway. Conversely we also heard many reports of how a positive ‘can do’ attitude of key general practice staff was instrumental in creating a willingness to embrace this novel approach and revise it as appropriate to individual and group practice. A systematic review of reflective practice in healthcare professionals identified frequency and tendency of reflection varying, but that it was successful in supporting sense-making [30], therefore we recommend that the importance of reflective practice is stressed in any future intervention.

## Conclusion

This intervention can be used to address known and emergent barriers to increasing chlamydia screening tests in family practice. Willingness of staff to adopt or adapt recommended strategies and resources including reminders, along with feedback on their efforts, was instrumental in embedding screening into practice. The findings from this study can impact practice by disseminating how the training, general practice screening rates, prompts and patient educational materials can overcome barriers to chlamydia screening and further inform what support the National Chlamydia Screening Programme offers. Table 6 shows the key determinants of success and actions that will facilitate them, as well as barriers identified by some participants and how other participants overcame these barriers during implementation of CIRT. Some general practices used extra interventions (such as postal invitations) that have previously been shown to be unsuccessful. In future training it may be useful to highlight these unsuccessful strategies. Reflective practice could be incorporated into follow up interventions.

**Table 1:** Interview participants by role and stratification by chlamydia screening rates post intervention

Staff role	High screening rates	Number of staff	Low screening rates	Number of staff
Doctor	Randomised	10	Randomised	10
	Not contactable/left practice/died	3	Not contactable/left practice/died	2
	Refused	2	Refused	4
	Interviewed	5	Interviewed	4
Nurse	Randomised	16	Randomised	16
	Not contactable/left practice/died	10	Not contactable/left practice/died	9
	Refused	0	Refused	3
	Interviewed	6	Interviewed	4
Receptionist	Randomised	6	Randomised	9
	Not contactable/left practice/died	0	Not contactable/left practice/died	2
	Refused	2	Refused	4
	Interviewed	4	Interviewed	3

**Table 2:** Coherence: was the purpose of the intervention clear

**About the workshop and value of inviting the whole team:** “*The support raised my awareness and it gave me different ways of approaching young people… I feel by including the reception teams that changed the surgery’s approach, I think it does one good to have one’s awareness raised*” (Nurse 04388) **Value of explaining epidemiology of chlamydia and how to perform the test:** “*I think it’s just having someone explain to you who needs what test… urine tests, swab tests, … and then what they actually practically do with it,… how long it will take for the results, … how it gets to the patient*” (General Practitioner 04380) “*That’s one of the things, because you get ideas about how you could manage it within this practice… being told what I can do, then I’ve got to go and try and embrace that, and get everybody else involved.”* (Nurse 03028) **A receptionists view:** “*I just think it’s* (chlamydia screening invitations) *more for a nurse more than receptionists but I suppose it’s a good idea for us to be aware a bit more. It’s just confidentiality isn’t it?* (Receptionist 04417)

**Table 3:** Cognitive Participation: staff engagement in training and general practice implementation

**How screening was promoted after the training:** “*It’s prompted us to have the kits and things on our desks…so we have greater awareness, to kind of dish it* (the screening tests) *out.*” (General Practitioner 04380) “W*e have posters around the surgery and cards that L* (a staff member) *will give them, we offer a condom service here, the c-cards, they will attach on* (to the) *cards* (information) *about the chlamydia service to those* (patients) *when they come for the free condoms,… and just generally in … consultations.*” (Nurse 04254) **Advantage of practitioners who are decision makers in general practice being at the training:** “*A couple of the lady doctors have come to a couple of the talks… and that always helps if you’ve actually got a GP there at that time*.” (Nurse 04243) **Value of being given possible scripts in the workshop to use with patients:** *“You just say something like: “We are a chlamydia screening surgery would you like one of these tests, we offer them to everybody under 25”…. so yes, yes that definitely helped*” (General Practitioner 03730) *“Let’s check your blood pressure; and you know you’re in the age group for a chlamydia screen have you had one in the last 12 months?”* (Non clinical 71) *“You just say something like: “We are a chlamydia screening surgery would you like one of these tests we offer them to everybody under 25” it did (*using the script*) so yes yes that definitely helped*” *(*General Practitioner 321) **Value of doing test immediately in the surgery:** “*Well I think we’ve begun to realise* (by) *talking to the chlamydia support team and talking amongst ourselves, that probably they* (the tests) *only ever really get done, if you actually make the person go and do the swab there and then and then bring it back”* (General Practitioner 03730) **Barrier, lack of interest in sexual health by all medical staff:** “*I’m the only, you know, GP who’s actually interested in gynaecology in this practice and two of the other female doctors aren’t interested in doing it* (or) *taking over from me in that respect when I, … retire or whatever, … I think you know* (it’s) *quite a general attitude,… not everyone* (GPs) *is interested in that* (screening) *or feels it’s important or relevant.… Whereas the nurses, a lot of them … are well-qualified in family planning … and they’re more approachable, you know people find it easier to talk to nurses often, than a ….male older doctor*.” (General Practitioner 03029) **Barrier: A practitioner talking about targets set nationally and not for individual general practices:** “*No there is no credible* (target)*… because first of all* (they Public Health England) *decide that a group of people in an area has got a certain type of infection,* (and) *promiscuity etc. and you say the average of the whole country is like that.* (But) *here it’s totally different from the practice in town, how can you* (set) *the same target for me and B practice* (in town) *you cannot do that*” (General Practitioner 03867) **Barrier: lack of confidentiality in reception area:** *"Because it's such a small practice, and because of confidentiality the problems of that* (chlamydia screening) *in a small practice, … and knowing everyone."* (Receptionist 04417)

**Table 4:** Collective Action: work undertaken to drive the intervention forward and sustain it

**Value of computer prompts:** *“Definitely* (computer) *pop ups, that that’s key almost, I think that that’s the biggest influence it’s had on me is pop ups, so it’s just automatic, because you know you’re looking there* (the computer screen) *anyway and then you see* (the prompt to) *offer chlamydia and you think ah yeah.”* (Nurse 03897) **Value of repeated contact from the Chlamydia Support Team:** *“Well they* (the Chlamydia Support Team) *just kept on with it, so they’ve kept contacting us, they’ve kept the interest going, kept coming up and it’s not just lets visit once and clear off, you know they’ve been on several occasions and that’s good”* (General Practitioner 04250) **Value of ongoing awareness raising in practice:** *“I’ve brought it up at a couple of meetings since then, and also introduced the little* (chlamydia patient invitation) *cards and things into reception… and said look… you really have to think about it. And as a new receptionist has come in; I’ve said* (to her) *you know (be) aware that these little chlamydia cards could also be attached to a prescription for the pills, or you know contraceptive type of prescriptions, or anybody coming to the* (reception) *hatch who looks to be in that sort of age group.”* (Nurse 04346) **Receptionist involvement:** *“Most of our screening is done sort of across the counter at the front desk, where and when we see a lot of new patients registering from the university which is quite local to us. We offer chlamydia screening routinely to that particular age group.”* (Receptionist 04430) **Barrier, perceived confidentiality issues:** “*She* (the chlamydia support worker*) told us that* (get the patient to complete screen in practice there and then) *but the practice isn’t big enough to do that, because there is only one toilet… that leads right out into the waiting room area and people would see people coming out with a packet… and they wouldn’t like to do that.*” (General Practitioner 03920) *“For one I would feel that I wouldn’t have the time,… so it would depend on how busy we were. And two I wouldn’t think it would be appropriate, it’s the sort of thing that somebody could complain about* (if) *you’re not keeping things confidential. So I would only ask if I knew there was no one behind them, even if it’s a woman coming in for a smear.”* (Receptionist 04207) **Barrier, lack of ability or will to develop a computer prompt:** *Well I suppose we could have done* [developed a prompt] *but there’s only three* (practices) *who have ours* (computer system) *they know* (how) *to deal with all those* (other) *sorts of computers but wouldn’t have known what to do* (with ours*).*” (General Practitioner 03920) *“Because there are thousands of them, people ignore them. We have a little box at the bottom with lists of prompts that this patients* (is) *due their blood pressure check, their thyroid check,* (lists 3 others*), cervical smears and … just as a general practitioner if I’m very honest with you I ignore them.”* (General Practitioner 04205) *“I think in reality probably half of time we just ignored it and thought I haven’t got time for that, but the other times it did trig your memory and you did it. We just changed to (*a new computer) *system a few months ago, and I don’t know whether it’s available on that, but we certainly don’t have it* (any prompts) *at the moment.”* (General Practitioner 03730) **Barrier, some receptionists uncomfortable discussing chlamydia screening:** *“I wouldn’t be comfortable sort of saying to a young person, when there’s a lot of say older people behind them, “would you want to do a chlamydia test” I just wouldn’t do it, so I don’t think it’s changed here in how we deal with the patients”* (Receptionist 04207) **Barrier: concern that patient will not welcome chlamydia screening offer:** *“I don’t think it’s appropriate to talk about these things* (chlamydia screening), *especially if you want to have a good relationship with your patients, it is just incredibly difficult.”* (General Practitioner 03867) **Barrier, staff uncomfortable raising screening in non-sexual health consultation:** “*I do however do quite a lot of telephone triage. Its slightly more difficult to think about chlamydia when you’re dealing with people with other health issues, be it sort of tonsillitis or you know whatever other issue they’ve got, it’s not always as easy on the phone to suddenly launch into sexual health question.*” (Nurse 04346) **Barrier, problems with access to chlamydia swabs:** *“Checking that nobody’s got any outdated swabs in their surgery, or you know the NAATS tubes, which sounds simple, but everybody (staff say) “I haven’t got any out of date ones” and then the following week you’ll get..a result back* (from the lab) *saying tube out of date, couldn’t do the test.”* (Nurse 04388) “*I think perhaps we have had* (some) *difficulty getting hold of the packs. I think if it* (were)*….a bit smoother ordering the packs and that sort of thing, … that would make us do more*” (Nurse 04254)

**Table 5:** Reflexive Monitoring: assessing impact of the work and making adjustments

**Testing data from the NCSP helps them to reflect on their testing rates and efforts:** *“(*Name of CSW) *certainly comes, and if our rates start dropping off, she starts sending us little emails “don’t forget chlamydia screens!”.* (General Practitioner 04250) **The more tests you do the easier it becomes:** “*You know it’s a bit like getting a wheel turning, you know as I said, you get more confident, you get more aware and it just becomes part of your daily work.”* (Nurse 04543) **By doing tests staff realise patients welcome being asked:** “*So it’s not… a taboo subject… this is what we do in the surgery, it’s standard practice “would you like a pack?” (*As) *Opposed… to the nurses being uncomfortable about it,* (or) *the young person feeling uncomfortable about it. It’s … built in, very much this is what we do, this is what we offer, are you interested and we haven’t had any negative feedback from that at all.”* (Nurse 04353) **Offering the test was quick:** “*I think it you can do it… within a minute, it doesn’t take a lot of time*” (Nurse 04254) *“It’s just a question of being organised really and keeping some* (kits) *available”* (General Practitioner 03029) **Value of putting a specific patient prompt for the next consultation:** *“I try and I do it as often as possible… if I do forget, if I know I’m bringing them back in, I’ll put a little alert on for myself to try and remember to do it the next time*” (Nurse 04346) “*even without the kind of financial incentive, I think if we recognise that it was a valid thing to do. I think given the initial potential training with regard to helping us to acknowledge that as an important thing to do. The logistics of this is, how you can do it, then I think we could have kind of just gone and flown with it as a result”* (General Practitioner 04387) **Barrier, time** *“It’s a ten minute appointment, its sometimes just not possible to even go there; we don’t even bother raising it because there’s just not enough time, because you’re running twenty minute late already*” (General Practitioner 04380) “*It does lengthen the consultation because you’ve got to be prepared”* (General Practitioner 03730) **Barrier, other priorities:** “*Because … when we discussed it and I mentioned that about reaching the targets and things and everyone just said we can’t do everything, and just you know do what you can, but we weren’t going* (to) *have a massive drive on this.*” (Nurse 3177)

**Table 6:** Key determinants of success and barriers identified and potential tools to overcome them

**Key determinants of success**	**Actions needed to facilitate them**
Attendance at workshop of all general practice staff	When workshops are organised ask and encourage all staff to attend.
Attendance at workshop by key staff able to drive forward screening	Ensure correct staff whose role it is to undertake, facilitate or lead screening attend the workshop.
Increased confidence to offer screening through using scripts and practice, so normalised into routine practice	Using scripts in the training, and encouragement post training – stressing that the process of offering tests will increase confidence and positive feedback from patients.
Provision of resources for flexible use	Ensure that resources provided are in a format that can be adapted for individual general practice needs.
Use of computer prompts	Raise this in training and when and how they are going to be used, and who is going to set them up.
Tests are performed in the general practice before the patient leaves	Stress importance in training and plan how this can be attained in each general practice.
Encouragement of reflective practice post workshop led by “chlamydia champion”	Encourage action planning and agree staff roles going forward. General practice lead to follow up with communications about successes and testing rates and sexual health news in general practice.
Feedback on screening efforts	Feedback general practice screening rates on a regular basis, and identify individual in the general practice to this to other staff.
**Barriers**	**Tools to overcome barrier**
Forgetting to offer chlamydia screen	Using computer prompts and posters, and identify individual to take this forward.
Not wishing to offer chlamydia screening in non-sexual health consultations	Staff changing the way they offer a chlamydia screen by stating that they are testing everyone aged between 15 and 24, and having a general prompt for all ages.
When sexual health is a low priority in the general practice	Ensure individual responsible for deciding if general practice will prioritise chlamydia screening attends the training. Having a designated general practice lead to drive screening. Ensure lead and general practice keep screening as a priority, and send around reminders and forward newsletters to all staff.
Perception of time involved in offering chlamydia screen, and monitoring screening rates	Use the scripts; stress that staff report that using them increases confidence and fluency in making the offer. Making sure there are posters to refer to, kits easily accessible and invitation cards available to give to patients. Suggest audits as part of professional development of all staff.
Patients not returning the chlamydia test	Feeding back that other general practice staff ask the patient to do the test prior to leaving the general practice, and this is what patients want too.
Ongoing perception that patients may be irritated if they are offered screening	Stress patients views in the training, lead to facilitate ongoing discussions in general practice around feedback from patients.
Lack of privacy in reception area to give out invitation cards	Stress in training that offer should be made to all in age group so not seen as judgemental by staff; and teach receptionists to use script saying "we are offering this test to all 15–24 year olds, read about it on this card."
Targets being too high for general practices with very low screening rates	Stress importance in the workshop training of discussing realistic screening rates for individual general practices with numbers and actions needed to attain them.

### Ethics

The study was approved by the Warwickshire Research Ethics Committee (REC Ref: 08/H1211/57)

## Appendix

### Post-intervention interview schedule

1. What is your role/job title in the practice?
2. How long have you worked here

We are interested in understanding how your own practice has been influenced, or not since your practice received the training and support offered by the SW Chlamydia Support team.

1. Can you start by describing your own approach to chlamydia screening?
2. Has the support given by xxx changed the way staff interact with young people- if so how, and with what effect on their practice?
3. Have you changed your practice regarding which patients you raise the possibility of having a chlamydia screen within consultations? If so which type of clinics/consultations/young people do you now target that you previously didn’t?
  - All consultations with age 15–24 year olds
  - Those known to be sexually active
  - Young people’s clinics
  - Other
4. Have you changed how you introduce the possibility of a chlamydia screen in a consultation? If so how and with what effects?
5. How confident are you to raise the possibility of a chlamydia screen in consultations- what has made it easier/more difficult?
6. Has your confidence about offering a chlamydia screen changed – if so what factors have influenced this?
7. Has working in this way (as above) affected the relationship you have with patients in these consultations?
8. Has working in this way meant you, or others, have more work to do?
  - Longer/shorter consultations
  - More/less work for others in the team
9. Have there been any changes in policies, training of staff or their roles to achieve higher screening rates?

### C: GP Practice Level

1. In your opinion, how important is it to public health and your patients that the rates of chlamydia screening set by the Government are met? How credible is the evidence for the targets?
2. How has the support from the chlamydia support worker (xxxx) influenced the approach of the practice to setting, recording and increasing the rates of chlamydia screens in this practice?
3. Has there been any change in how offers, actual screens and results of screens are recorded in the practice?
4. Has there been any change in how the practice’s rates of screens and results are used in the practice?
  (recorded, reported/feedback to whom, what actions?)
5. Has there been any feedback on the practice’s rates of screening from the PCT- if so was this helpful or not? How might this feedback be improved?
6. Do you think the practice has increased its chlamydia screening rates over the past year or not- if so why? What might have made it (even) more successful?
7. Has the practice changed who is involved in offering chlamydia screening in the past year -if so how and with what effects?
  Prompts:
  - Information displays/drop boxes or other patient initiated methods
  - Reception staff activities
  - Nursing staff consultations
  - GPs consultations
  - Other

### Overall Impact

1. Overall, has the training and support from SW Chlamydia Support and how the practice has responded improved, or had negative impacts on how the practice operates?
2. How important do you think the training and support was to achieving the changes you and your practice made? What might you have done differently?
3. Would you recommend the training to other practices?
4. What aspects of the training and support would you recommend to be used by other PCTs?
  Training session, Feedback of screening rates, Newsletters, Posters, Reception cards, Prompts, Website.
5. What aspects of the training and support would you not recommend to be used by other PCTs?
6. Anything else to add?
